# Fetal hemothorax due to intrapleural rupture of a small anterior thoracic lymphatic malformation: a case report

**DOI:** 10.1186/s13256-026-06618-3

**Published:** 2026-10-05

**Authors:** Haruna Kawaguchi, Katsuya Hirata, Wakako Ichida, Sayaka Fukuda, Masahiro Zenitani, Naruhiko Takahashi, Shusaku Hayashi

**Affiliations:** 1 Department of Maternal Fetal Medicine, Osaka Women’s and Children’s Hospital, 840, Murodo-Chou, Izumi, Osaka 594-1101 Japan; 2 Department of Neonatal Medicine, Osaka Women’s and Children’s Hospital, 840, Murodo-Chou, Izumi, Osaka 594-1101 Japan; 3 Department of Radiology, Osaka Women’s and Children’s Hospital, Osaka, Japan; 4 Department of Pediatric Surgery, Osaka Women’s and Children’s Hospital, Osaka, Japan; 5 Present Address: Department of Obstetrics, Kochi Health Sciences Center, 1-2125 Ike, Kochi City, Kochi 781-8555 Japan

**Keywords:** Fetal hemothorax, Lymphatic malformation, Hydrops fetalis, Magnetic resonance imaging, Case report

## Abstract

**Background:**

Fetal pleural effusion is usually chylous, whereas hemothorax is considerably rarer. Trauma, iatrogenic complications, and rupture of intrathoracic tumors have been reported as causes of fetal hemothorax. Lymphatic malformations often enlarge during the fetal period; however, these benign lesions may regress spontaneously after birth or be managed with sclerotherapy. Accurate prenatal diagnosis is therefore crucial for determining whether fetal intervention or postnatal management is the most appropriate strategy. Here, we present a rare case of a fetus with hemothorax presumably caused by the rupture of a small anterior chest wall lymphatic malformation that progressed to hydrops fetalis.

**Case presentation:**

A Japanese woman in her 30 s (gravida 2, para 1) was referred at 26 weeks of gestation after bloody pleural fluid was obtained by thoracentesis at another hospital. On admission, ultrasonography showed right pleural effusion without ascites or subcutaneous edema. Additionally, there was a small heterogeneous echogenic region in the anterior chest wall without detectable blood flow, although its clinical significance was initially unclear. Fetal magnetic resonance imaging demonstrated pleural fluid with signal characteristics consistent with hemothorax and identified a small anterior chest wall cystic lesion, suggestive of a lymphatic malformation. Because the pleural effusion was hemorrhagic, thoracoamniotic shunting was avoided owing to concerns regarding rebleeding and potential worsening of fetal anemia. At 27 weeks and 6 days, an emergency cesarean section was performed for worsening hydrops. The infant required immediate intubation, chest drainage, and blood transfusion. Postnatal imaging confirmed an anterior chest wall lymphatic malformation extending toward the mediastinum. The lesion did not require surgical or sclerosing therapy and showed spontaneous regression during follow-up. Neurodevelopmental assessment was within the normal range at a corrected age of 18 months.

**Conclusion:**

This case demonstrated the importance of detailed imaging studies, including magnetic resonance imaging, for the diagnosis of pleural effusion and small anterior chest lesions. In cases of tumor-associated fetal hemothorax caused by benign lesions such as lymphatic malformations, early delivery followed by neonatal management may represent a reasonable alternative to fetal intervention.

## Background

Fetal pleural effusion can occur for various reasons. The majority of cases are idiopathic chylothorax caused by lymphatic leakage [1, 2], whereas other etiologies include chromosomal abnormalities, congenital heart disease, fetal infections, and immune or nonimmune hydrops. Fetal hemothorax is extremely rare, with reported aetiologies including trauma [3], shunt-related iatrogenic causes [4], and rupture of intrathoracic tumors [5]. Lymphatic malformations often enlarge during the fetal period; however, these are benign congenital lesions that can undergo spontaneous regression after birth or can be managed medically with sclerotherapy. Intracystic hemorrhage has been frequently reported [6]. However, rupture of a lymphatic malformation into the pleural cavity during the fetal period has rarely been documented. Herein, we present a rare case of a fetus with a small anterior chest wall lymphatic malformation complicated by a hemothorax leading to hydrops fetalis, and describe successful perinatal management and favorable outcomes.

## Case presentation

The patient was a Japanese woman in her 30 s (gravida 2, para 1) with a spontaneous conception. Her medical history was notable for pulmonary sarcoidosis, which required no treatment. There was no notable family medical history. Until 24 weeks of gestation, routine ultrasound revealed no pleural effusion or other abnormal findings. At 26 weeks and 2 days of gestation, right pleural effusion was detected. At 26 weeks and 4 days, ultrasonography at the referring hospital showed right pleural effusion without ascites or subcutaneous edema. It also revealed a heterogeneous echogenic region without blood flow to the anterior chest wall. Middle cerebral artery peak systolic velocity (MCA-PSV) was 1.3 multiples of the median (MoM), showing no elevation. Thoracentesis was performed at the referring hospital on the same day, and bloody pleural fluid was obtained with a hemoglobin (Hb) of 10.5 g/dL and hematocrit (Ht) of 30.9%. The following day, the effusion reaccumulated, and the patient was referred to our hospital.

**Fig. 1:**
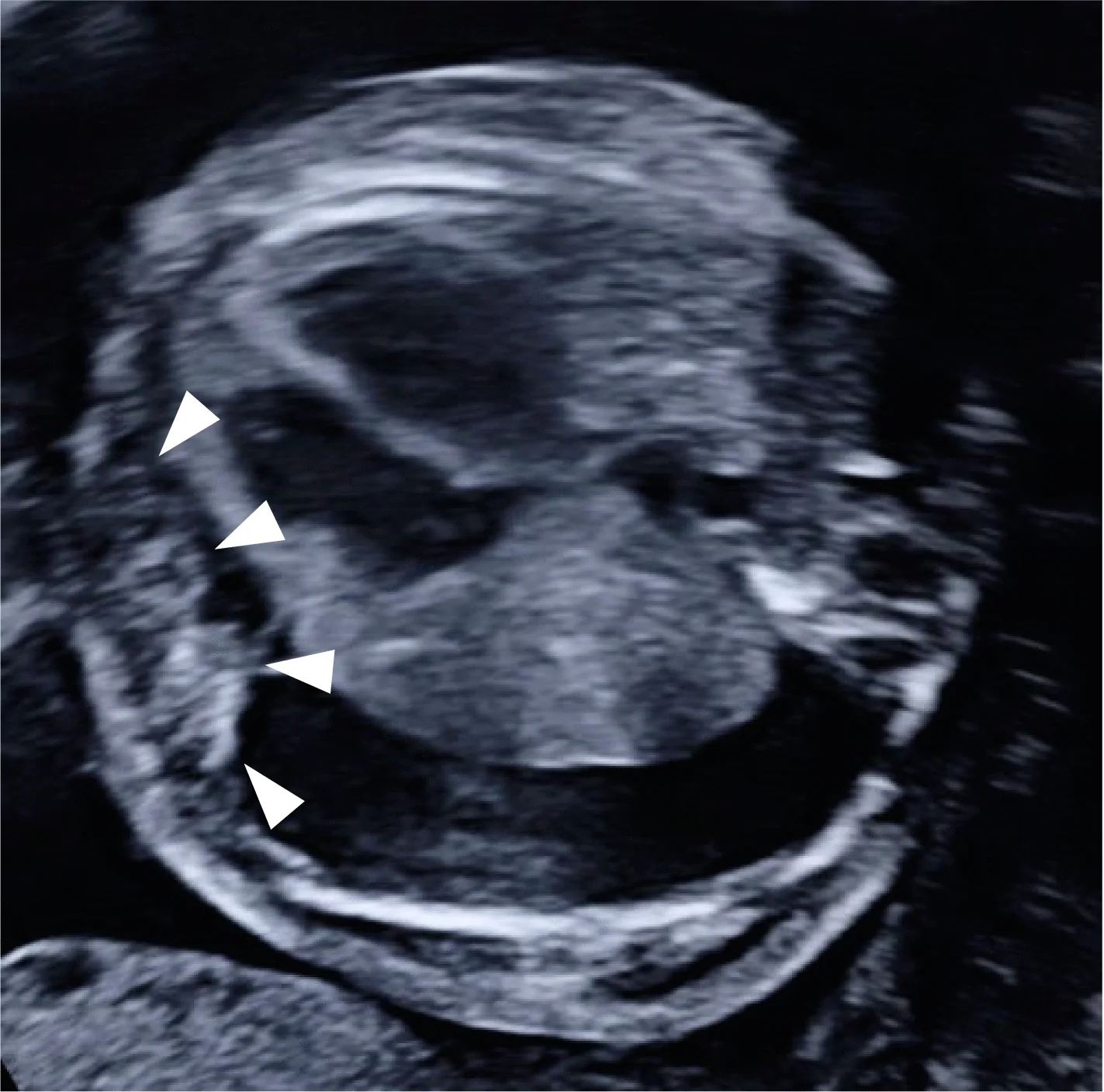
Ultrasonographic findings at first visit. Low-echoic fluid is observed in the fetal right pleural cavity. The area indicated by the white triangles represents the lesion in the anterior chest wall

**Fig. 2:**
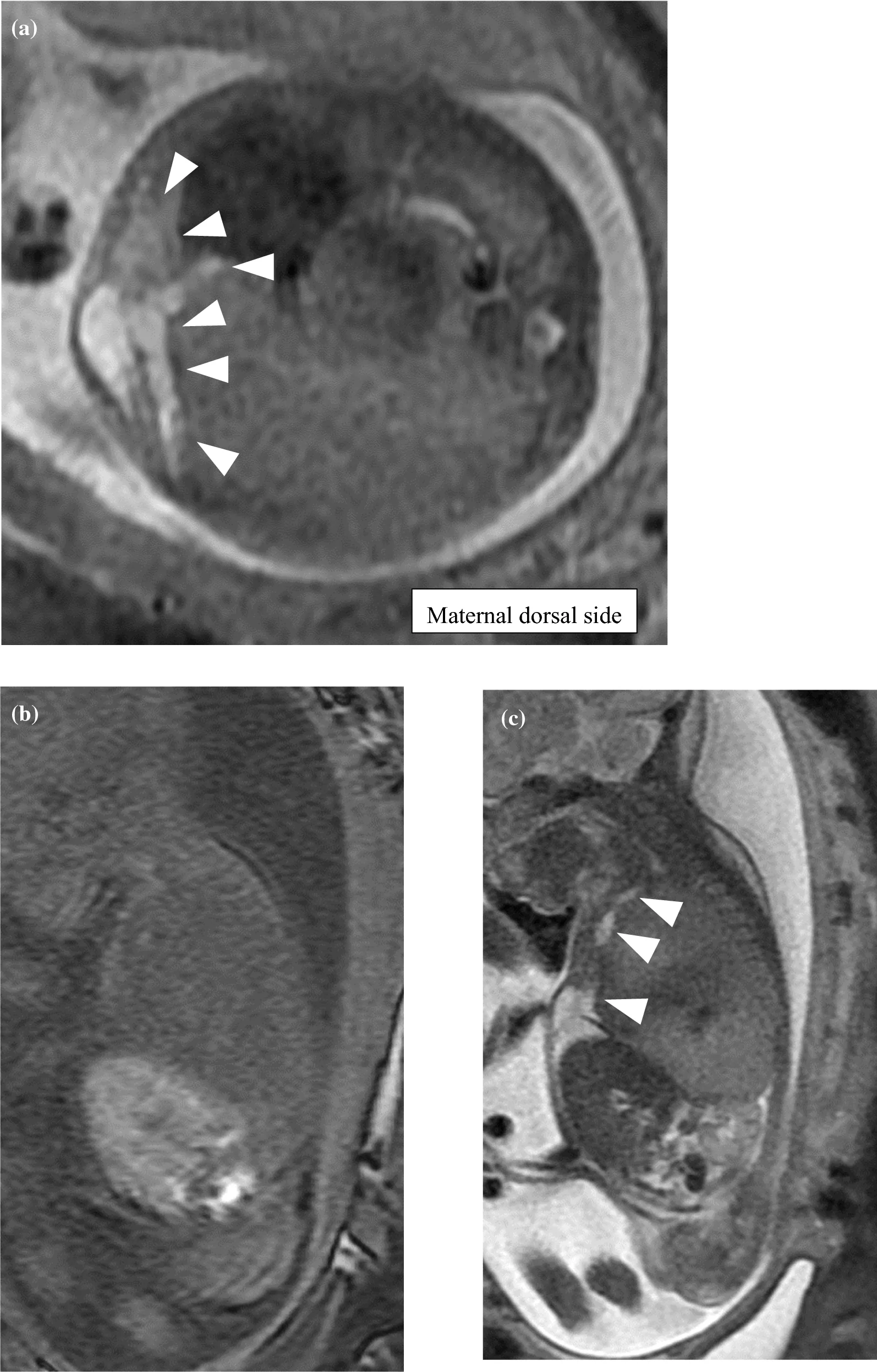
Fetal magnetic resonance imaging. **a** Axial T2: weighted chest image. **b** Sagittal T1: weighted chest image. **c** Sagittal T2: weighted chest image. A multilocular cystic lesion is identified in the anterior chest wall (white triangles). The right pleural effusion shows a slightly higher signal intensity than the amniotic fluid on T1-weighted images and a lower signal intensity on T2-weighted images

**Fig. 3:**
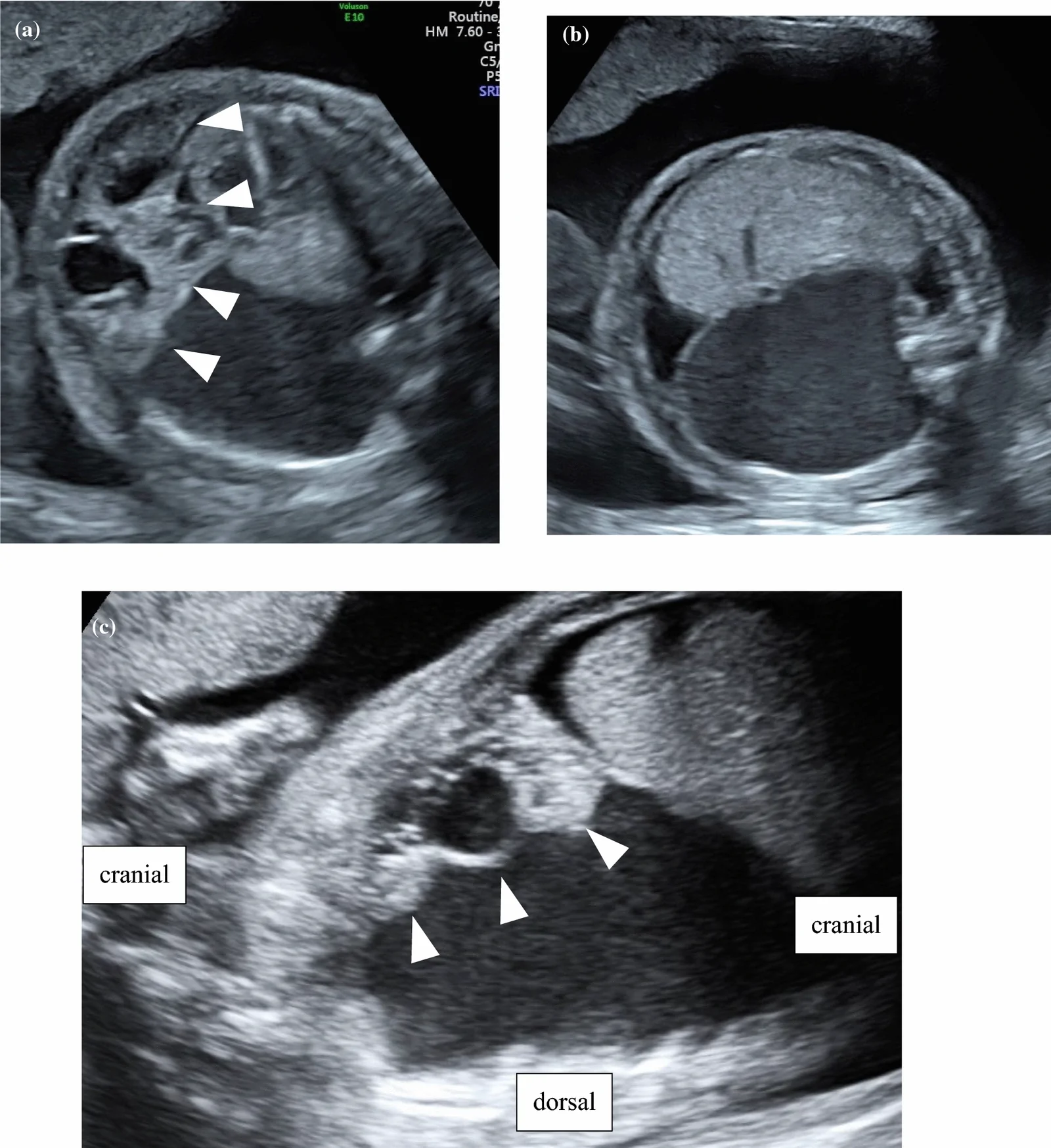
Ultrasonographic findings at 27 weeks and 5 days of gestation. **a**, **b** Axial chest views. **c** Sagittal chest view. The mass is enlarged in the anterior chest wall (white triangles). Right pleural effusion demonstrates higher echogenicity than ascites

**Fig. 4:**
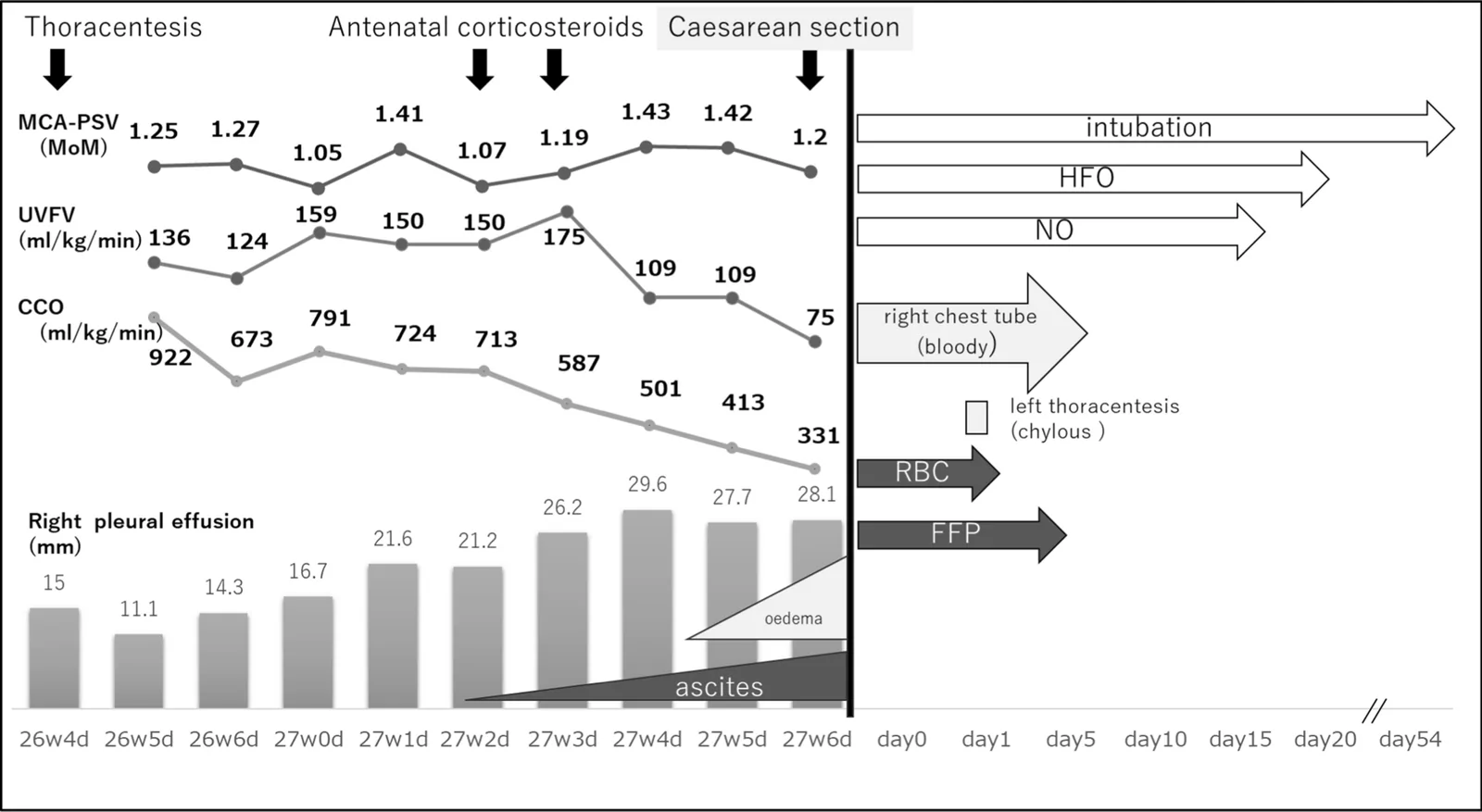
Timeline of the antenatal and postnatal clinical course. MCA-PSV remained within the upper limit of the normal range, whereas UVFV and CCO decreased as the right-sided pleural effusion increased. Ascites and subcutaneous edema subsequently developed, consistent with progression to hydrops fetalis. After birth, a right-sided chest tube was placed and remained in situ until day 5 of life. Left-sided pleural effusion was treated with a single thoracentesis, with no subsequent reaccumulation. The infant required mechanical ventilation until day 54 of life. *GW* gestational week, *MCA PSV(MoM)* middle cerebral artery peak systolic velocity (multiples of the median), *CCO* combined cardiac output, *UVFV* umbilical vein flow volume, *HFO* high-frequency oscillatory ventilation, *NO* nitric oxide, *RBC* red blood cell transfusion, *FFP* fresh frozen plasma

On initial examination at our center, right pleural effusion was confirmed, whereas left pleural effusion, ascites, and subcutaneous edema were absent. A 26×9.5 mm mass containing small cystic components was observed in the fetal anterior chest wall (Fig. 1). Fetal magnetic resonance imaging (MRI) was performed at 27 weeks and 2 days of gestation. A multilocular cystic lesion was observed in the anterior chest wall. The right pleural cavity showed a slightly higher signal intensity than the amniotic fluid on T1-weighted images and a lower signal intensity than the amniotic fluid on T2-weighted images. The lower signal intensity on the maternal dorsal side suggested the presence of blood components within the right pleural cavity. (Fig. 2a–c). These findings were highly suggestive of rupture of the fetal anterior chest wall lymphatic malformation into the pleural cavity. Serial ultrasound examination showed enlargement of the anterior chest mass and a gradual increase in the right pleural effusion (Fig. 3c). From 27 weeks and 2 days, mild ascites appeared, followed by abdominal subcutaneous edema at 27 weeks and 4 days. The fluid component in the right pleural cavity exhibited higher echogenicity than ascites (Fig. 3a, b). By 27 weeks and 6 days, the hydrops had progressed with scalp, thoracic, and abdominal edema, and left-sided pleural effusion had appeared. Doppler evaluation revealed no qualitative abnormalities in the waveforms of the umbilical artery or ductus venosus. MCA-PSV remained within the normal range (1.0–1.4 MoM). Figure 4 shows the ultrasound findings during the prenatal course. As the pleural effusion volume increased, the cardiothoracic ratio gradually decreased, and both the total cardiac output and umbilical vein blood flow progressively declined.

A multidisciplinary team comprising obstetricians, neonatologists, pediatric surgeons, and radiologists concluded that the benefits of iatrogenic preterm birth combined with neonatal intensive care outweighed those of continuing the pregnancy. Following this discussion, the parents provided informed consent for the proposed management. Antenatal corticosteroids were administered at 27 weeks and 2 days, and at 27 weeks and 3 days of gestation. Owing to the progression of fetal hydrops, an emergency cesarean section was performed at 27 weeks and 6 days, and a female infant weighing 1,375 g was delivered with Apgar scores of 3 and 3 at 1 and 5 min, respectively. The infant was intubated and received high-frequency oscillatory ventilation with supplemental inhaled nitric oxide (NO). Owing to persistent ventilatory failure caused by right-sided pleural effusion, a right-sided chest tube was inserted at 13 min of life to drain the bloody pleural fluid. Pleural fluid analysis showed Hb of 4.0 g/dL and Ht of 13.4%, which were lower than the prenatal thoracentesis values, with cytology revealing lymphocyte predominance (62%). Blood tests indicated anemia with Hb 8.2 g/dL and Ht 25.7%, necessitating blood transfusion. The reticulocyte count was elevated (206‰), suggesting increased haematopoiesis in response to fetal anemia. A peripheral venous catheter was inserted, and continuous infusion of dopamine and dobutamine was initiated.

**Fig. 5:**
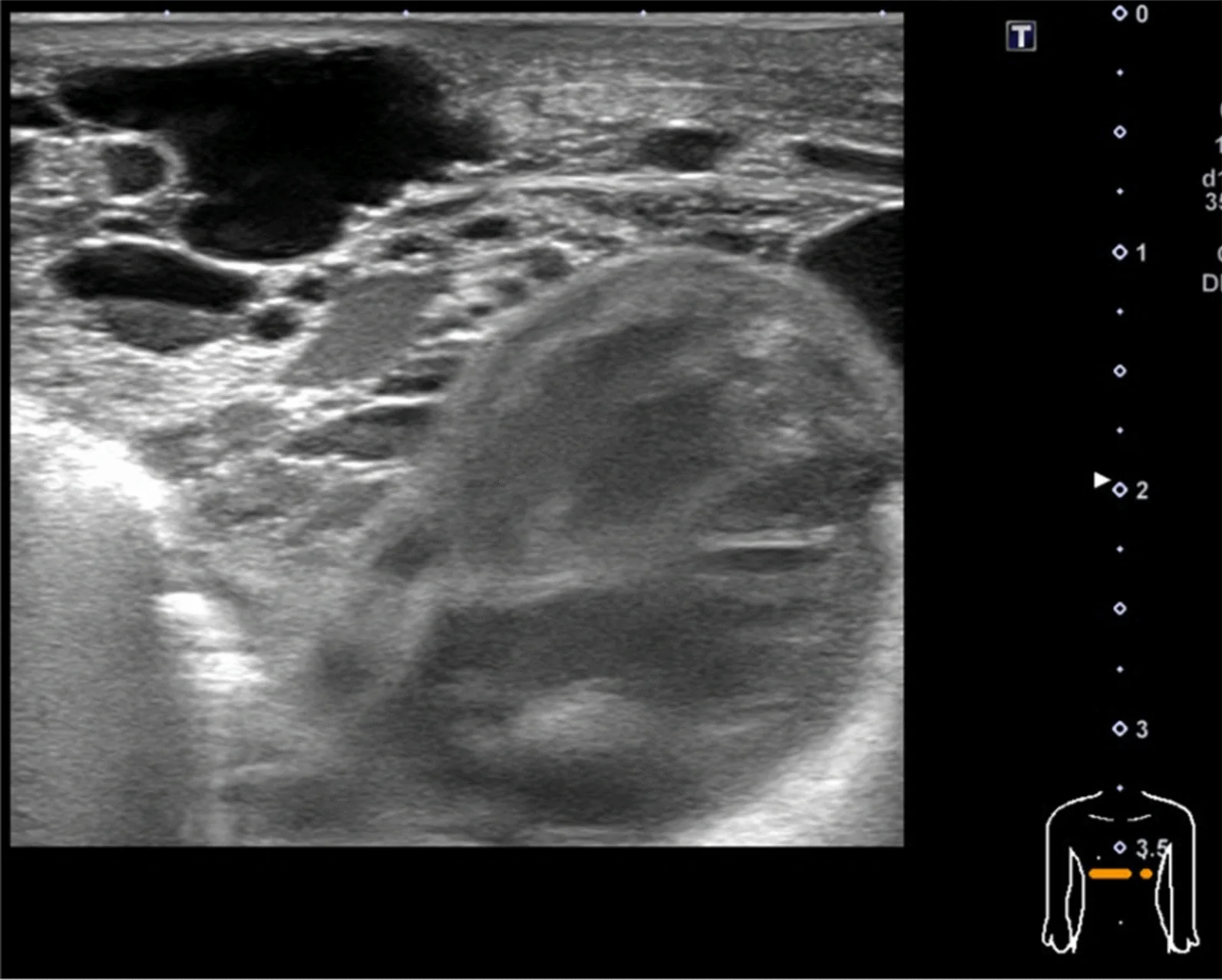
Early postnatal ultrasonography. Echocardiography revealed an enlarged cystic mass in the anterior chest wall. A fluid level was observed inside the cyst, suggestive of hemorrhagic content

**Fig. 6:**
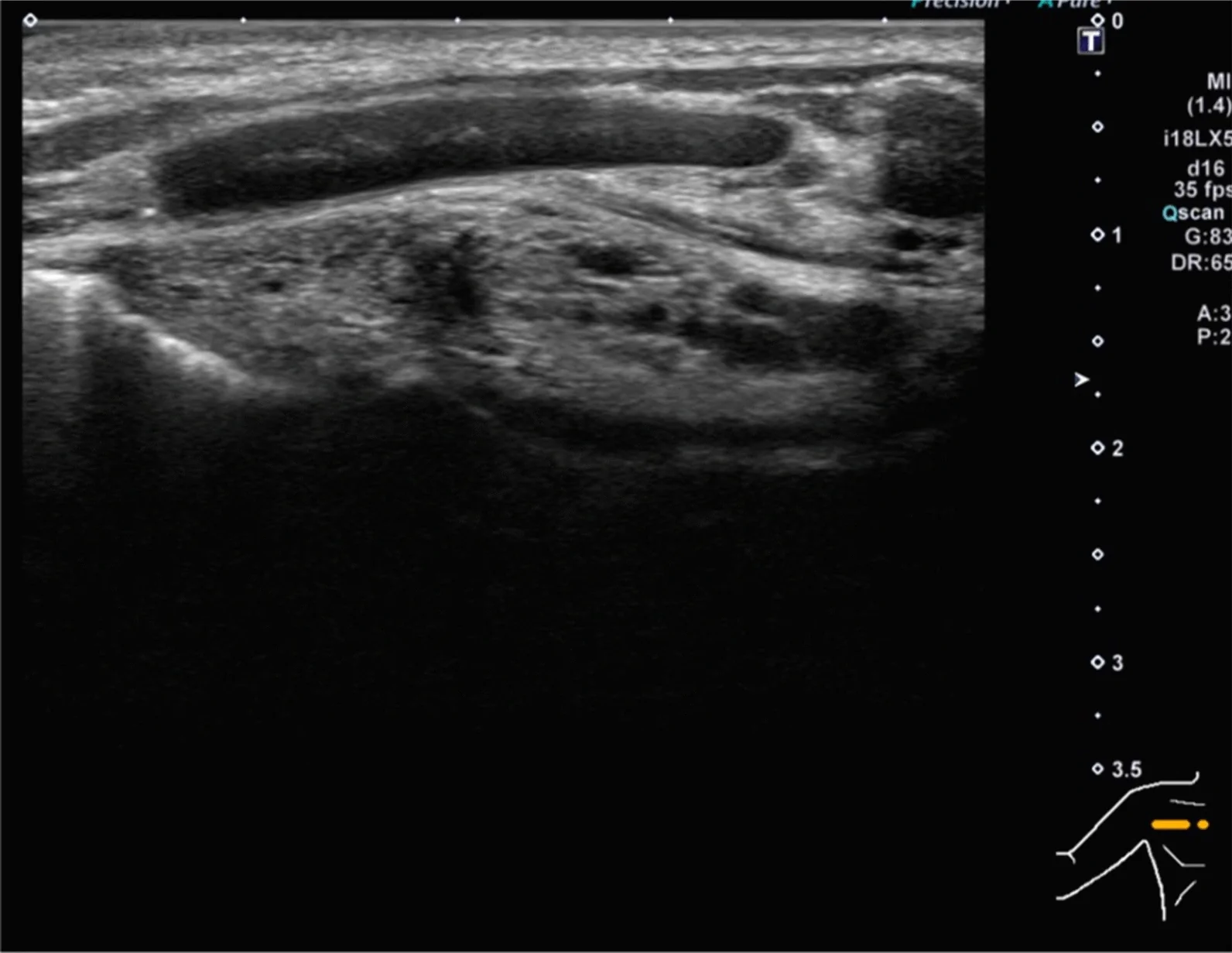
Ultrasonographic findings at 1 year and 6 months of age. The lymphatic malformation has regressed spontaneously, without the need for intervention

Ultrasonography revealed a lymphatic malformation extending from the anterior chest wall to the mediastinum (Fig. 5). On day 1 of life, 50 mL of chylous fluid was drained via left thoracentesis with no subsequent accumulation. The right chest tube was removed on day 5 of life, and no recurrence was observed thereafter, allowing the discontinuation of NO administration. Extubation occurred on day 54 of life, and oxygen supplementation was discontinued on day 96. Hemorrhage was observed within the cystic component of the lymphatic malformation after birth; however, there was no recurrence of the hemothorax. The lesion showed a tendency toward spontaneous regression, with mild enlargement and atrophy. By 1 year and 8 months of age, it had resolved without requiring intervention (Fig. 6). No unexpected adverse events occurred due to early delivery and transition to neonatal care. The infant underwent developmental assessment using the Kyoto Scale of Psychological Development, a standardized tool widely used in Japan to evaluate cognitive, language, and motor development in young children [7]. At a corrected age of 18 months, the full-scale developmental quotient was 88, which was within the normal developmental range.

## Discussion and conclusion

Fetal pleural effusion is almost always chylous, and hemothorax is rare. Lymphatic malformation is a benign congenital lesion that most commonly occurs in the neck, with only approximately 10% of cases occurring within the thoracic cavity. Hemorrhage within cysts has been reported in approximately 9% of lymphatic malformations [6]; however, there have been no reports of pleural cavity rupture. In the present case, fetal thoracentesis yielded markedly bloody pleural fluid (Hb 10.5 g/dL and Ht 30.9%), supporting the diagnosis of hemothorax. Iatrogenic bleeding related to thoracentesis was considered. However, nearly all of the aspirated pleural fluid was markedly bloody, prenatal MRI showed signal characteristics suggestive of hemorrhagic pleural fluid, and bloody pleural fluid was again drained immediately after birth. Furthermore, postnatal imaging demonstrated hemorrhage within the cystic component of the lymphatic malformation, further supporting an association between the lymphatic malformation and the hemothorax. Taken together, these findings supported the presence of pre-existing fetal hemothorax rather than simple blood contamination during thoracentesis, although an iatrogenic contribution could not be completely excluded. In the fetal diagnosis of this case, MRI was an important tool, in addition to ultrasound. First, regarding the nature of the pleural effusion, ultrasonography showed it to be hypoechoic and could only be identified as a liquid component. MRI demonstrated signal intensities different from those of amniotic fluid, suggesting the presence of hemorrhagic pleural fluid. Furthermore, the anterior chest mass appeared cystic on the initial ultrasound examination; however, its thinness and small size made it difficult to determine whether it was truly a mass. However, MRI clearly localized the mass, and its shape revealed it to be a lymphatic malformation.

Fetal pleural effusion gradually increased, but the MCA-PSV value did not exceed 1.5 MoM. Therefore, we hypothesized that the fetal pleural effusion contained both bloody and lymphatic components. The Hb/Ht values in the right pleural effusion confirmed after birth were lower than those during the fetal period, and, as suspected, lymph fluid was also present. The MCA-PSV was within the normal range; however, the newborn presented with anemia at birth and required a blood transfusion. The reason for the discrepancy between the MCA-PSV and neonatal hemoglobin level remains uncertain. Severe pleural effusion and altered fetal hemodynamics may have affected the MCA-PSV response. Reticulocytosis is a compensatory mechanism reflecting enhanced fetal hematopoietic function. This case involved rapid progression to fetal hydrops following thoracentesis, prompting a discussion on whether to remove the effusion via thoracentesis or to perform a thoracoamniotic shunt.

The efficacy of thoracoamniotic shunting has been reported for primary fetal hydrothorax [8]. However, its efficacy for fetal hemothorax has not been established. Moreover, hemothorax has been reported as a complication following thoracoamniotic shunt placement [4]. The clinical course suggested possible involvement of both the hemothorax and lymphothorax; however, fetal thoracoamniotic shunting was not selected because of concern regarding further bleeding and potential exacerbation of fetal anemia. On the other hand, fetal transfusion is considered an option when an increase in MCA-PSV is observed; however, no increase was detected, and the procedure was not performed. When fetal thoracic amniotic shunting was not selected, fetal hydrops was considered to have progressed, leading to imminent fetal death. As an alternative, switching to neonatal treatment can be considered; however, the early delivery of fetal hydrops results in a considerably poor prognosis for neonates [9]. The key feature in this case was the underlying condition of a small lymphatic malformation. Lymphatic malformations often enlarge during the fetal period, but are benign lesions that can undergo spontaneous regression after birth or be managed medically with sclerotherapy [6, 10, 11]. Therefore, we deemed neonatal treatment following early delivery a viable option. As a limitation of this case report, since a thoracoamniotic shunting was not performed, the possibility that fetal hydrops improved and the gestational period was extended cannot be ruled out. On the other hand, prenatal sclerotherapy with intracystic administration of OK-432 has been reported to reduce fetal lymphatic malformations [12]. In addition, sirolimus has recently been reported as a potential prenatal treatment for large fetal neck and facial lymphatic malformations via maternal administration through the placenta [13]. In the present case, the lymphatic malformation gradually increased in size; however, the cystic components remained small. The cause of hydrops fetalis was circulatory failure due to hemothorax resulting from rupture of the pleural cavity, not compression by the mass itself. Therefore, intrauterine sclerotherapy and pharmacological tumor reduction with sirolimus were not considered appropriate treatment options in this case.

This case represented a rare instance of fetal hemothorax, presumably caused by rupture of an anterior chest wall lymphatic malformation, leading to hydrops fetalis. Considering the underlying condition, switching to neonatal treatment was feasible in this case. The decision to proceed with delivery before further progression of hydrops fetalis contributed to the successful outcome. This case demonstrated the importance of detailed imaging studies, including MRI, for diagnosing pleural effusion and small anterior chest masses. Accurate imaging enabled the selection of appropriate treatments for the infant.

## Data Availability

Not applicable.

## Abbreviations

MRI: Magnetic resonance imaging
MCA-PSV: Middle cerebral artery peak systolic velocity
MOM: Multiples of the median
Hb: Hemoglobin
Ht: Hematocrit
NO: Nitric oxide
GW: Gestational week
CCO: Combined cardiac output
UVFV: Umbilical vein flow volume
